# Bones geometric morphometrics illustrate 10th millennium cal. BP domestication of autochthonous Cypriot wild boar (*Sus scrofa circeus* nov. ssp)

**DOI:** 10.1038/s41598-021-90933-w

**Published:** 2021-06-01

**Authors:** Thomas Cucchi, Auriale Domont, Hugo Harbers, Allowen Evin, Roger Alcàntara Fors, Maria Saña, Charlotte Leduc, Aurélie Guidez, Anne Bridault, Hitomi Hongo, Max Price, Joris Peters, François Briois, Jean Guilaine, Jean-Denis Vigne

**Affiliations:** 1 Archéozoologie, Archéobotanique: Sociétés, Pratiques et Environnements, AASPE UMR 7209, CNRS/Muséum National d’Histoire Naturelle, Paris, France; 2 ISEM, University of Montpellier, Montpellier, France; 3 Autonomous University of Barcelona, Barcelona, Spain; 4 Trajectoires, de la Sédentarisation à l’État, UMR 8215, Maison de l’Archéologie et de l’Ethnologie, 21 Allée de l’Université, 92000 Nanterre, France; 5 Archimède, Archéologie et Histoire Ancienne: Méditerranée Europe, UMR 7044, Université de Strasboug, Strasboug, France; 6 ArScAn, Equipe Archéologies Environnementales, UMR 7041, CNRS, Maison de l’Archéologie et de l’Ethnologie, 21 Allée de l’Université, 92000 Nanterre, France; 7 Department of Evolutionary Studies of Biosystems, School of Advanced Sciences, Graduate University for Advanced Studies, Shonan Village, Hayama, Kanagawa 240-0193 Japan; 8 Department of Materials Science and Engineering, Massachusetts Institute of Technology, 77 Massachusetts Avenue, Cambridge, MA 02143 USA; 9 ArchaeoBioCenter and Institute of Palaeoanatomy, Domestication Research and the History of Veterinary Medicine, LMU Munich, 80539 Munich, Germany; 10 SNSB, State Collection of Anthropology and Palaeoanatomy, 80333 Munich, Germany; 11 EHESS, UMR 5608, Travaux et Recherches Archéologiques sur les Cultures, les Espaces et les Sociétés (TRACES), Université Jean Jaurès, Toulouse, France; 12 Collège de France, 11, Place Marcelin-Berthelot, 75005 Paris, France

**Keywords:** Evolution, Zoology

## Abstract

Epipaleolithic hunter-gatherers from the Near East introduced wild boars (*Sus scrofa*) to Cyprus, with the Early Pre-Pottery Neolithic (PPN) settlers hunting the wild descendants of these boars. However, the geographic origin of the Cypriot boar and how they were integrated into the earliest forms of pig husbandry remain unsolved. Here, we present data on 11,000 to 9000 cal. BP *Sus scrofa* from the PPN sites of Klimonas and Shillourokambos. We compared them to contemporaneous populations from the Near East and to Neolithic and modern populations in Corsica, exploring their origin and evolution using biosystematic signals from molar teeth and heel bones (calcanei), using 2D and 3D geometric morphometrics. We found that the Cypriot PPN lineage of *Sus scrofa* originates from the Northern Levant. Yet, their phenotypic idiosyncrasy suggest that they evolved into an insular sub-species that we named *Sus scrofa circeus,* referring to Circe, the metamorphosis goddess that changed Ulysses companions into pigs. The phenotypic homogeneity among PPNA Klimonas wild boars and managed populations of PPNB Shillourokambos suggests that local domestication has been undertaken on the endemic *S*. *s. circeus*, strengthening the idea that Cyprus was integrated into the core region of animal domestication.

## Introduction

During the Younger Dryas cold spell, Epipalaeolithic people introduced wild boars (*Sus scrofa*) to Cyprus, where *Sus scrofa* remains have been direct dated from the site of Akrotiri-Aetokremnos to around 12,000 cal. BP^1^. By that time, dwarf hippos and elephants, the main megafauna endemic to Cyprus, had been extinct for several centuries^2^. The anthropogenic introduction of wild boars was therefore probably intended to replenish the island’s ecological niche with suitable large game^3^. According to preliminary studies, this translocated population rapidly developed insular syndromes, marked by a 10–16% reduction in body size^4–6^. Suids remained the sole ungulate on the island until the early Pre-Pottery Neolithic B (PPNB), around 10,500 cal. BP. In fact, these small wild boar were the only large game from the PPNA settlements of Asprokremnos and Klimonas, dated to 10,800 cal. BP, contributing 95% of the vertebrate remains collected from these sites^7^. According to the kill-off patterns documented for Klimonas, these small wild boars were hunted by targeting females and their young^7^. Five centuries later, PPNB communities reached Cyprus and brought domestic goats and cattle, Mesopotamian fallow deer and finally sheep^3,8^. Palaeodemographic studies of suid remains from PPNB Shillourokambos suggest that the exploitation of suids in the course of the PPNB shifted from hunting strategies to seasonal slaughtering, typical of Mediterranean herding^9^. Traditional osteometric analyses have tentatively suggested that these PPNB communities engaged in the management and domestication of endemic Cypriot wild boar, but could not exclude that the villagers also introduced some domestic pigs from the Continent, along with their other domestic ungulates^4,10^. Cypriot wild boar obviously went extinct long before the Common Era^11^.

Due to poor DNA preservation in faunal specimens from arid contexts^12^, we considered the biosystematics resolution of dental forms^13,14^, together with the ecophenotypic resolutions of the heel bone (calcaneus)^15^ to investigate the origin and evolution of PPN suids in Cyprus. The taxonomic resolution of dental shape at the intraspecific level^16–18^ allowed us to disentangle the evolutionary components of insularity and domestication in ancient Cypriot wild boar phenotype. Additionally, the phylogenetic signature of dental forms in mammals^13,14,19,20^ facilitated investigation regarding the origins of PPN suids in Cyprus via comparison with contemporaneous Continental populations^21^. Finally, the ecophenotypic plasticity of the calcaneus in mammals^22,23^, recently evidenced to capture the anthropogenic control of wild boar locomotion behaviour^15^, enabled us to detect changes in suid mobility from free-ranging to penning. Palaeodemographic information from reconstructed kill-off patterns provided complementary markers to understand long-term human–suid interaction in early Neolithic Cyprus.

## Materials

Geometric Morphometrics (GM) were performed on standardized 2D images of the second (M/2) and third (M/3) lower molars, collected from modern (Table 1) and archaeological (Table 2) samples of wild boars and domestic pigs. A total of 86 modern *Sus scrofa* specimens were analysed, including a variety of Continental wild boar populations from the Eastern and Western Mediterranean Basin (Turkey, Syria, Algeria, Tunisia, and Morocco) and insular populations from Corsica and Sardinia including 28 hunted wild boars, 32 corsican pigs landrace (*U nustrale*) and 7 hunted wild/feral hybrids. The Corsican and Sardinian wild boar have an anthropogenic origin, descending from feralized pigs introduced by Neolithic communities during the 7th millennium BC^24^. The ancestry of these Neolithic pigs is traceable to a South-West Asian *Sus scrofa* lineage^25,26^. The diversity of populations (wild, feral, domestic and hybrids) found in these islands provides a relevant comparative reference to explore the diversity of the PPN Cypriot *Sus scrofa*.

**Table 1:** Modern samples of wild and domestic *Sus scrofa* used for dental Geometric morphometrics.

Modern samples			
Origin	Taxa	N M/2	N M/3
Corsica	Domestic pig	32	18
Corsica	Wild boar	19	5
Corsica	Wild crossed	7	3
Sardinia	Wild boar	9	9
Syria	Wild boar	3	1
Turkey	Wild boar	10	6
Northern Africa	Wild boar	6	5
	Total	86	47

**Table 2:** Archaeological samples of *Sus scrofa* selected for dental Geometric morphometrics.

Archaeological samples					
Site	Period	Date cal. BP	Code	N M/2	N M/3
Hallan Çemi	PPNA	12,500–10,800	HLC	4	8
Çayönü			CAY-PPNA	5	8
Göbekli			GOB	1	1
Ain Ghazal	PPNB	10,800–8700/8200	AGT	2	1
Çayönü			CAY-PPNB	20	17
Nevali Çori			NVC	4	5
Tell Halula			HAL	9	6
Gürcütepe			GUR	1	1
Domuztepe	PN	9000/8200–5300	DOM	2	8
Çayönü			CAY-PN	4	4
Araguina	Middle Neolithic	6300–5300	COR-ARA	2	0
Terrina IV	Late Neolithic	5300–4500	COR-TER	5	5
Klimonas	PPNA	11,100–10,600	KLI	33	26
Shillourokambos middle A	PPNB	9600–9300	SHI-A	25	12
Shillourokambos middle B			SHI-B	2	5
Total				119	104

**Figure 1:**
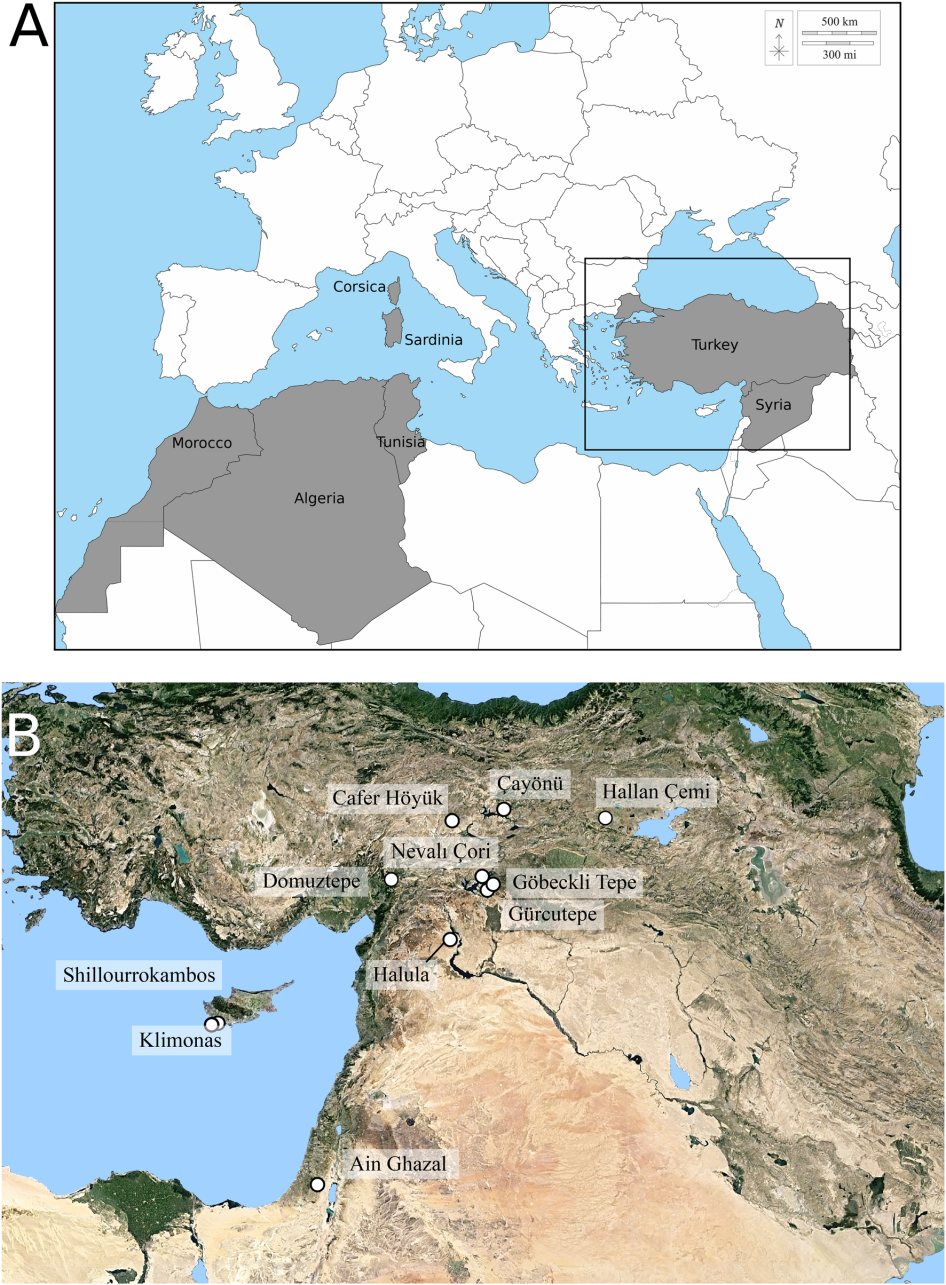
**(A)** Geographic provenance of the extant wild boars samples. **(B)** Localization of the PPN sites with *Sus scrofa* samples. Maps adapted from D-map (https://d-maps.com) by T. Cucchi and D.G. Kuriyama. Figure generated by TC with Inkscape 1.0.2.

A total of 119 archaeological *Sus scrofa* were sampled from ten PPN sites in the Upper Tigris and Euphrates River Basins and Cyprus (Fig. 1), as well as two Neolithic sites from Corsica, Araguina Sennola and Terrina IV (Vigne 1988). The latter were used as proxy for early insular domestic forms (Table 1). The sample size for the PPN Cypriot *Sus scrofa* is large, with more than 30 individuals from PPNA Klimonas and more than 20 individuals from the PPNB middle phases A and B of Shillourokambos (Late Cypro-PPNB) (Table 1). Unfortunately, important pre- and post-depositional alterations prevented the sampling of *Sus scrofa* dental remains from the earliest PPNB occupation at Shillourokambos.

The sample size for the calcaneus 3D shape analysis was smaller due to the analytical requirement for the fused calcaneal tubercle (Harbers et al. 2020). The necessity for a fused proximal epiphysis relates directly to the biomechanics of the ankle joint. Three extensor muscles attached either directly or through the Achilles tendon to the calcaneal tubercle enable the lever function of the calcaneus, thus facilitating movement in the mammal’s hind limb. In this respect, the analysis had to be restricted to two fused specimens from Cypro*-*PPNA Klimonas and six from Late Cypro*-*PPNB Shillourokambos. The comparative collection constituting the calcaneus shape analysis baseline includes both modern and archaeological specimens. Modern wild boars include 28 specimens from several extant populations across Western Europe, in addition to 24 experimental wild boars taken from the wild after weaning and being raised under two systems of reduced mobility: 100 m^2^ stall versus a 3000 m^2^ pen, until two years old (see Harbers et al. 2020 for details). Modern pigs include 22 specimens from European landraces and 5 specimens of the Corsican landrace. Archaeological specimens include 28 Mesolithic wild boars as proxy for the phenotypic variation of European wild boars prior to the Neolithic transition and subsequent dispersal of Near Eastern domestic lineages to Western Europe. These specimens come from five 10,000–8000 cal. BP Mesolithic sites in France: Ranchot, Arconciel^27^, Noyen-sur-Seine^28^, Téviec^29,30^ and Gazel^31^.

## Methods

The morphometric analysis of both phenotypic markers (molar and calcaneus) relies on a GM approach which allows size and shape components to be distinguished^32^. We used 2D and 3D Cartesian coordinates on homologous anatomical points (landmarks) and constructed points on the curves and surfaces of homologous elements (semi- or sliding landmarks), to capture the morphological complexity of teeth and bones with accuracy. It also allows the geometry of these forms to be preserved and visualized graphically throughout statistical analyses.

**Figure 2:**
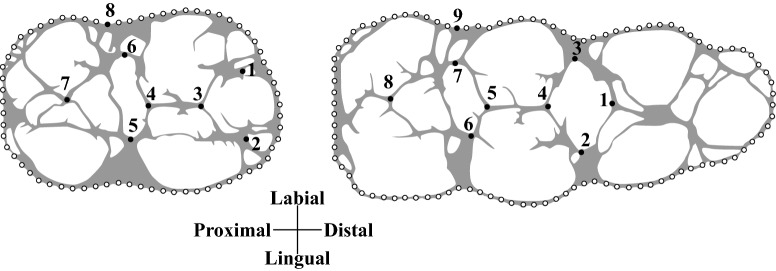
GM protocol for lower M/2 and M/3.

The M/2 and M/3 forms were analysed using 2D GM from standardized pictures of molar occlusal views (Fig. 2). The occlusal morphology of the molar was captured using landmarks on the occlusal surface associated with semilandmarks along the external outline of the crown, following previous studies^17,18,33–36^ using TPS dig 2.20^37^. We made some minor changes to the previous protocol by using only one landmark on the external outline of the crown. M/2 and M/3 have 8 and 9 landmarks, respectively, with the last landmarks being used as the starting point of the outline curves from which the equidistant semilandmarks were extracted: 67 for the M/2 and 99 for the M/3.

The form of the calcaneus was captured with 3D GM from virtual 3D objects reconstructed by the segmentation of either a medical CT scan using Avizo 8.1 for modern comparatives or with photogrammetry for the archaeological specimens using Agisoft PhotoScan Standard (Version 1.2.6), retrieved from http://www.agisoft.com/downloads/installer/. The 3D GM of the calcaneus associates homologous landmarks on anatomical points with semilandmarks on curves and surfaces for the most comprehensive acquisition of the complex calcaneus shape (see Harbers et al. 2020 for methodological details). The 3D landmarks and semilandmarks were collected using Morpho-package.R^38^.

The Cartesian coordinates of 2D (x,y) and 3D (x,y,z) points were standardized before statistical analyses with a Procrustes superimposition using Geomorph^39^ and Morpho^38^ packages. Procrustes superimposition removes information of size (scale), position and orientation from the set of landmarks and semilandmarks’ Cartesian coordinates characterizing each specimen. Procrustes coordinates obtained after the alignment are the shape variables used is subsequent statistical analysis. Centroid Size (C Size) for each specimen was computed as the square root of the sum of squared distances between the centre of gravity (centroid) and each landmark. The Procrustes coordinates and centroid sizes are the shape and size variables of the statistical analyses below. To obtain the form (size + shape) dataset one have to concatenate the log(C size) vector with the Procrustes coordinates in a single matrix submitted to the multivariate statistical analyses^40^.

### Statistical analyses

Differences in size variation among modern and archaeological samples was tested with an Analysis of Variance (ANOVA) and graphically displayed with box plots.

The presence of several potential populations among the PPN samples of Cyprus was investigated with a density estimation via a Gaussian finite mixture analysis of the log CSize variation with the MCLUST version 3 package^41^.

Comparative shape analysis was performed with multivariate statistics. We first investigated the allometric component of shape variation with a covariation between centroid size and shape using a Procrustes ANOVA, taking into account the differences between the populations with a Multivariate Analysis of Covariance (MANCOVA).

Shape differences between groups were tested with a factorial MANOVA while the shape differentiation was visualized with Linear Discriminant Analysis (LDA), computed on a reduced dataset after Principal Component Analysis (PCA) on the Procrustes coordinates. PCA scores accounting for 95% of the variance were used for the LDA. The percentage of correct classification in each population groups were computed with a two-fold cross validation over 10,000 iterations.

Dental form similarities and dissimilarities among population samples have been displayed using an unrooted phenotypic tree computed with a neighbour-joining tree algorithm (ade4 package) based on the Euclidean distance between the mean shapes of each group sample.

To predict the ecomorphological status (free ranging or controlled) of the PPNB Cypriot wild boars based on the calcaneus shape variation, we used the k-nearest neighbour (k-NN) machine learning algorithm from the class package in MASS. This non-parametric approach relies on a training set of known classes: (1) modern and archaeological hunted wild boars, (2) experimental captive wild boars and (3) landrace pigs. The k*-*NN classification of the archaeological specimens relies on the class membership of the majority of its closest neighbours. To define the k number of the nearest neighbour, we used the conventional approach of the square root of *N*.

All statistical analyses were performed in R^42^.

## Results

### Molar form variation in modern insular and continental populations of wild and domestic *Sus scrofa*

**Figure 3:**
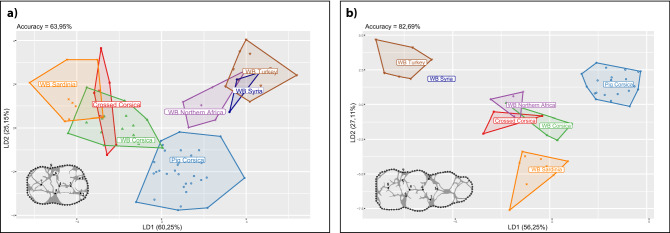
*Sus scrofa* molar form differences in extant continental and insular wild (WB) and domestic (Pig) populations. The percentage of each group’s correct classification performed after cross-validation (accuracy) is provided: **(a)** CVA displaying discriminant axes LD1 and LD2 for the M/2 form variation; **(b)** CVA displaying discriminant axes LD1 and LD2 for the M/3 form variation.

We found significant differences in molar shape (MANOVA M/2: *df* = 6, *F* = 4.77, *p* < 0.001; MANOVA M/3: *df* = 6, *F* = 2.69, *p* < 0.005) between the modern wild and domestic *Sus scrofa* and a common biosystematics pattern between the M/2s and M/3s, despite some disparities due to sample size differences (Fig. 3).

This analysis is able to discriminate domestic from hunted specimens within Western Mediterranean insular *Sus scrofa* populations. Additionally, for both molars, the insular populations of wild boars and hybrid specimens cluster as a homogeneous insular morphogroup, phenotypically closer to the continental population of Northern Africa than to continental populations of the Eastern Mediterranean.

We found significant molar size differences between extant *Sus scrofa* populations when grouped by wild/domestic status and geographic location (ANOVA Procrustes: *df* = 1, *Rsq* = 0.085, *F* = 4.43, *p* < 0.001). These size differences introduced a significant allometric component explaining 8.5% of the overall shape variation (*p* < 0.001). However, these allometric trends in dental shape variation are shared among modern populations (MANCOVA population factor: centroid size; *F* = − 0.4889, *p* = 0.698) and do not interfere with the molar shape differentiation of the *Sus scrofa* populations. For this reason, we analysed the form dataset for M/2 and M/3 in order to access greater biosystematic resolution and disentangle the effects of insularity and domestication in the phenotypic make-up of the PPN Cypriot *Sus scrofa*.

### Size and shape variation in PPN *Sus scrofa*

**Figure 4:**
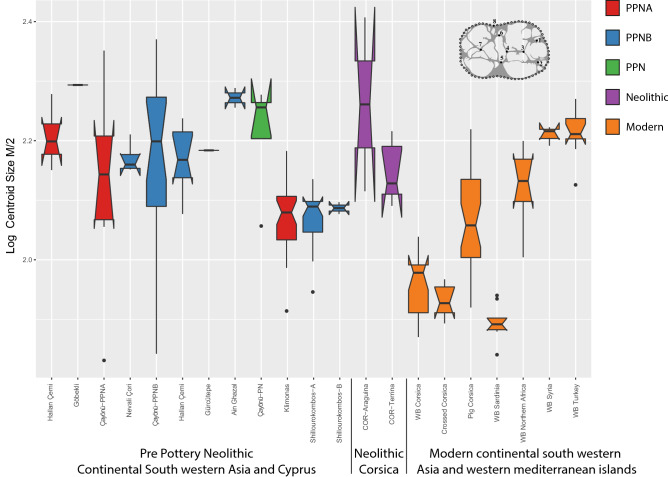
M/2 log(C Size) variation in extant and archaeological *Sus scrofa* displayed with box plots. The boxes represent 75% of the variation, the bold bar is the median and the vertical line the minimum and maximum extremities. Outliers are visualized by points.

**Figure 5:**
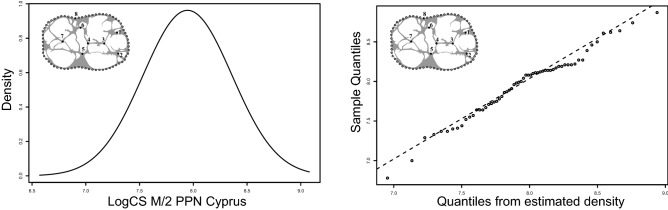
Density estimation via Gaussian Mixture modelling of the M/2 log centroid size in PPN *Sus scrofa* from Klimonas and Shillourokambos.

Molar size differences across extant and archaeological samples (ANOVA M/2: *df* = 20, *F* = 12.32, *p* < 0.0001) are displayed for M/2 in Fig. 4. Compared to current populations, the size of PPN Cypriot suids is similar to Corsican domestic landrace, smaller than the continental wild boar of Northern Africa and the Near East (Turkey and Syria), but larger than modern Corsican and Sardinian wild boars*.* The latter are the smallest *Sus scrofa* currently in the Mediterranean Basin and are considered as a separate sub-species named *S. scrofa meridionalis* (Groves et al., 2007). The variation in size of the Cyprus suids is quite low, when compared to PPN *Sus scrofa* from the Levant and Neolithic Corsica. The homogenous molar size variation between Klimonas and Shillourokambos *Sus scrofa* is typical of a single population as shown by the Gaussian modelling (Fig. 5).

**Figure 6:**
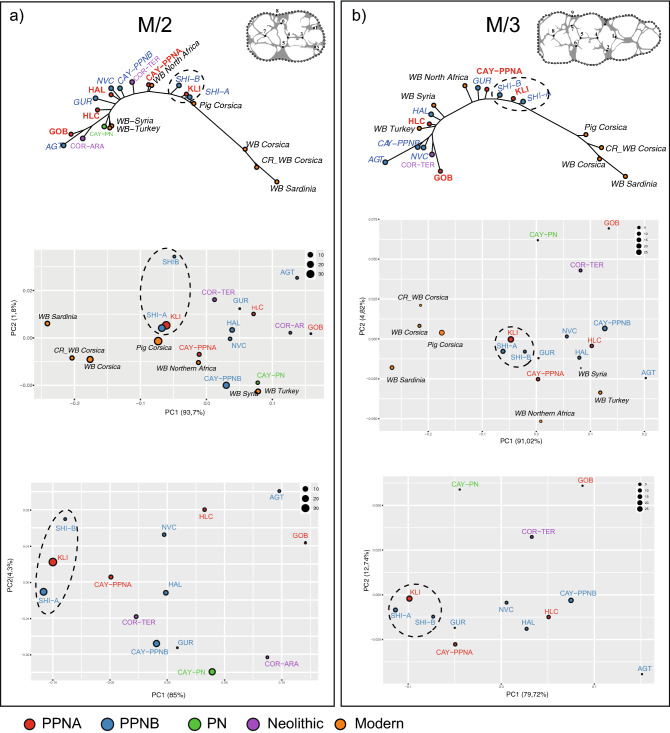
Patterns of similarities and dissimilarities in mean dental forms of M/2 **(a)** and M/3 **(b)** among modern and archaeological Mediterranean *Sus scrofa* displayed with an unrooted neighbour-joining tree (above) and a PCA (middle and bottom). PCA on archaeological mean forms only are displayed in the bottom panel.

The patterns of dental form similarity and dissimilarity between extant and archaeological samples (Fig. 6a,b), sheds light on three important points. First, Corsico–Sardinian *Sus scrofa* are phenotypically very distant from both extant and archaeological continental *Sus scrofa*. Secondly, PPN *Sus scrofa* from Klimonas and Shillourokombos middle phases A and B are phenotypically similar and diverge from continental PPN *Sus scrofa* towards the Corsico-Sardinian morphotype. Finally, Klimonas and Shillourokombos show greater dental similarities with PPNA Çayönü (for both M2 and M3) and Gürcütepe (just for the M3).

### Calcaneus size and shape variation of the Cypriot PPN *Sus scrofa*

**Figure 7:**
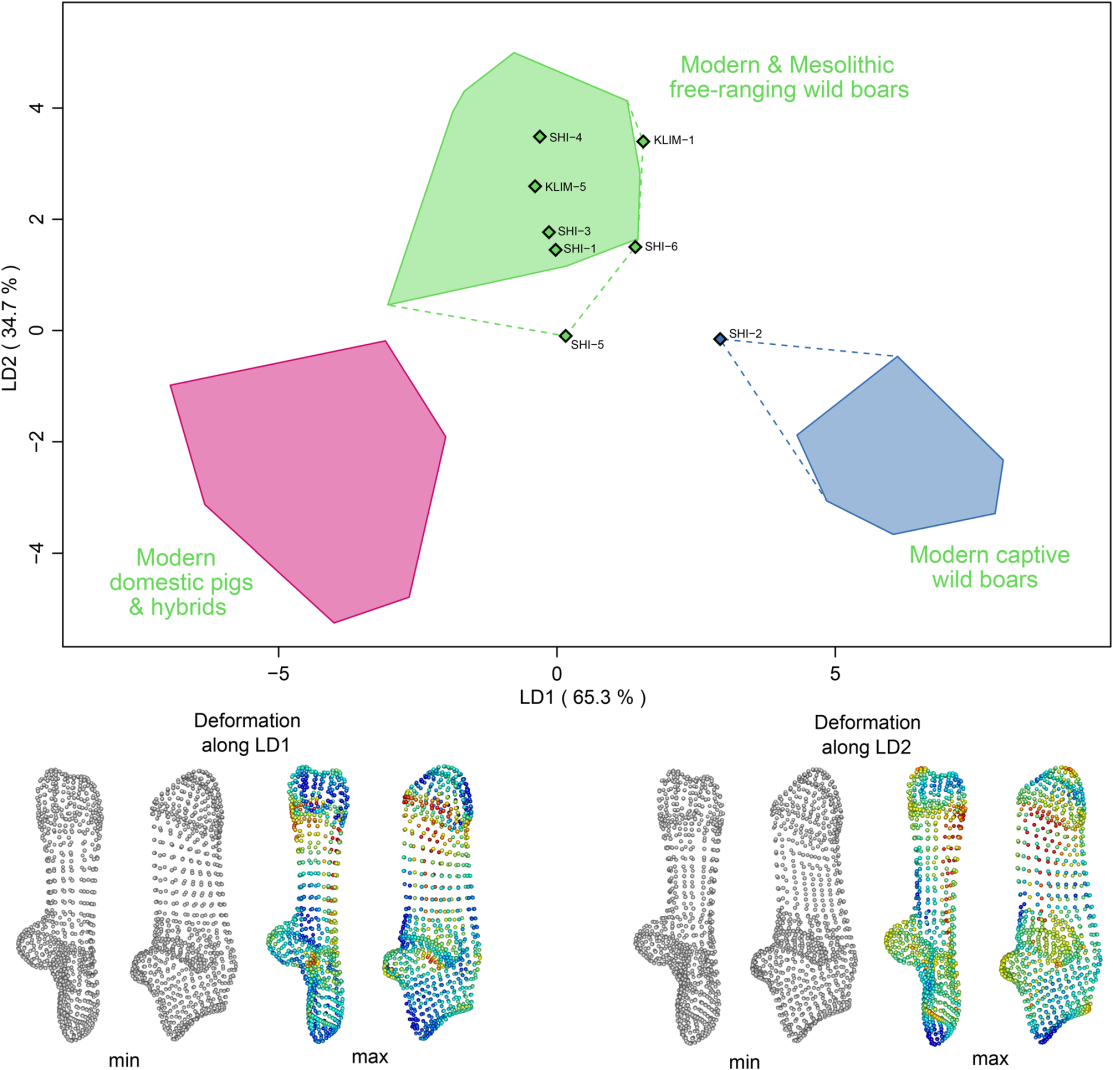
**(a)** Discriminant shape space of *Sus scrofa* calcaneus computed from modern and Mesolithic wild boars as well as extant domestic pigs. Modern wild boars include individuals born in the wild but raised in captivity until the age of 2 years. Klimonas (KLIM) and Shillourokombos (SHI) specimens have been projected onto this discriminant shape space and their membership to three morphogroups: free-ranging wild boars, captive wild boars and domestic pigs has been assessed by k-NN algorithm. Deformation along the two discriminant axes are displayed using heatmaps where greater change are shown in red.

The calcaneus 3D shapes of the PPN specimens from Klimonas and Shillourokambos were projected in the discriminant 3D shape space built using a modern and archaeological comparative dataset (Fig. 7). In this discriminant morpho-space, we observed significant divergence between hunted Mesolithic and extant wild boars, captive wild boars, and domestic pigs resulting from the last 200 years of selective breeding (Harbers et al. 2020). The projection of the PPN specimens from Cyprus in this morpho-space found that all Klimonas and most Shillourokambos *Sus scrofa* are phenotypically similar to wild boars hunted in their natural habitat, without anthropogenic mobility constraints. However, according to k-NN prediction, a single specimen from Shillourokambos fits the norm of reaction of a wild boar whose locomotor behaviour has been controlled though captivity.

## Discussion

### Molar form variation: biomarker of anthropogenic species evolution

The multivariate analysis of molar form variation in indigenous Mediterranean *Sus scrofa* enables continental and insular populations to be distinguished. Within Corsican *Sus scrofa*, it is also able to differentiate populations recently affected by anthropogenic selective processes of domestication. These results confirm the relevance of molar form variation as a phenotypic marker to understand to what extent the “island effect,” the domestication process, and the possible introduction of domestic pigs during the PPNB has impacted suid morphology in Cyprus. The extant domestic *Sus scrofa* from Corsica are landraces adapted to extensive husbandry practiced for several millennia^43^. Since Corsican swine herders have recently cross-bred these animals with improved continental breeds, such as Duroc or Large White^44^ in order to increase meat production^45^, we cannot fully disregard the possibility that recent gene flow contributed to the observed dichotomy between wild boar and domestic pigs. One might argue, therefore, that such a clear divergence between domestic and wild populations would not be observable in the archaeological dental record of early domestication. However, GM analyses of *Sus scrofa* molar series from Neolithic China has demonstrated a clear divergence of early Neolithic populations from the wild morphotypes, followed by an acceleration of this divergence by the middle Neolithic^17,34^, implying that the variation of molar morphology could be a reliable determinant of the early process of domestication, at least when some form of reproductive isolation is in place.

Among the Corsican populations of *Sus scrofa,* hybrids do not display an intermediate dental phenotype but rather a dental morphology similar to wild boar. This stands in contrast to our previous studies of domestic, wild and hybrid suids from other contexts^18^. One potential explanation is that these hunted hybrids have a very low level of hybridization, limiting the amount of gene flow from domestic pigs. Another suggestion is that hybrids with a closer phenotypic proximity to the wild forms would have had greater fitness. These hypotheses, however, need to be explored further.

### Insular evolution and the origin of the wild boar of Circe (*Sus scrofa circeus* nov. ssp)

The PPN Cypriot *Sus scrofa* display a homogeneous and idiosyncratic dental morphology. This morphological divergence from the continental relatives follows an evolutionary trajectory similar to that of the insular populations of wild boars from Corsica and Sardinia, considered as part of the *Sus scrofa meridionalis* subspecies (Groves 2007). These two observations support the hypothesis that PPN *Sus scrofa* could also be considered as a now extinct^11^ wild boar subspecies indigenous to Cyprus. We propose to name this sub-species: the wild boar of Circe: *Sus scrofa circeus*. This name refers to Circe, the Greek goddess of *Metamorphosis*, who turned Ulysses’ companions into swines in order to populate her island with pigs.

According to evolutionary models of insular mammals^46–48^, the idiosyncrasy of *Sus scrofa circeus* was probably the result of rapid adaptive change in a comparably short time interval, leaving no trace of intermediate phenotypes in the archaeological record. Indeed, the earliest remains of *Sus scrofa circeus* discovered in the Epipaleolithic deposits of Akrotiri-Aetokremnos are distal phalanges exhibiting a decreased size due to the insular syndrome, encompassing both size reduction and allometric decrease of extremity size^1^.

The fast evolutionary change of *Sus scrofa circeus* is indicative of a very small-sized founder population. Genetically isolated from their mainland relatives of continental South-West Asia, the founding population went through both genetic drift and adaptive radiation induced by the constraints of a novel insular ecological niche, as previously observed in another anthropogenic insular mammalian species, the Orkney vole *Microtus arvalis orcadensis*^46,49^. Homogeneity in the dental form of Cypriot *Sus scrofa*, from the 11th millennium cal. BP until the end of the 10th millennium cal. BP, is also consistent with the insular model, which postulates that rapid morphological change is followed by a stasis in absence of major environmental crises^46,50^.

The wild boars introduced to Cyprus by Natufian/Khiamian foraging communities ca. 12,000 years ago were probably the founder population of *Sus scrofa circeus*. Their phenotype remained stable until at least the end of the PPNB ca. 9000 years ago. *Sus scrofa circeus* represents the first anthropogenic populations of insular ungulates in the Mediterranean Basin, about two millennia before the start of *Sus* domestication (Vigne, 1999).

### Continental origin of *Sus scrofa circeus*

A consequence of the rapid evolutionary change in insular mammals is the morphological divergence from their mainland ancestors, complicating identification of their geographic origin^46,50^. However, dental form analyses have shown that the morphological divergence of *Sus scrofa circeus* did not reach the extent of insular endemism observed in current *Sus scrofa meridionalis* from Corsica and Sardinia. It is therefore still possible to observe some phenotypic similarities between PPN Cypriot and PPNA Çayönü *Sus scrofa*. Considering the phylogenetic signal preserved in the dental form of ungulates^13,14^, our results suggest that the continental source of endemic Cypriot wild boars may have been located in South-East Anatolia. Obviously, these results must be further supported as our dataset for the Levant is limited to the Late PPN site of Ain Ghazal. To confirm this initial assessment, future work should ideally include earlier sites such as Final Natufian Ain Mallaha (Bridault, ongoing research) and PPN Jericho and Tell Aswad.

The Euphrates and Tigris River basins, however, represent regions where we have the earliest evidence for cultural control and early management (and later domestication) of *Sus scrofa* populations^51–56^. In addition, there is decidedly more archaeological evidence connecting PPN Cyprus with Anatolia and the Northern Levant than with the Southern Levant. For instance, obsidian blades of East and Central Anatolian origin were found at both Klimonas and Shillourokambos^57,58^. Many cultural traits recorded at these sites are similar to the ones of the Northern Levant and Anatolian PPN sites^6,59^. The introduction of domestic goat and cattle c. 10,500 cal. BP occurred at a time when they are only known in the Northern Levant^3,60^. The Mesopotamian fallow deer is also likely to have been introduced from there^8^. Last but not least, the Epipaleolithic lithic assemblages from Cyprus, which are contemporaneous with the introduction of wild boar, display similarities with the Anatolian ones^61^. It is therefore more likely that the Epipaleolithic foragers that introduced the first managed wild boars to Cyprus originated from a geographic area that stretched from the foothills of the Eastern Taurus up to the Anatolian coastal region. Our results suggest that the morphological affinities of the PPN Cypriot wild boar provides additional evidence for strong cultural connections between Cyprus, Southeast Anatolia and the Northern Levant starting in Epipaleolithic times and continuing well into the PPN.

### Local domestication of endemic Cypriot wild boars during the PPNB

Comparative 3D analysis of the *Sus scrofa* calcaneus concluded that all the specimens from Klimonas displayed a phenotypic variation fitting the reaction norm of wild boars behaving in their natural habitat^15^. Therefore, humans must have acquired wild boar meat through hunting, a scenario supported by the kill-off pattern and the abundance of flint arrow heads at this PPNA village^7^.

Most *Sus scrofa* from the middle B phase of Shillourokambos display the same ecophenotypic variation as wild boars caught in the wild, with the exception of a single specimen whose shape is consistent with that of a wild boar raised in captivity^15^. It is worth noting that the calcaneus fuses at around 36 months, meaning that the animals included in our study are older than 97.8% of the 36 suid individuals attested in the age profile observed for this phase at Shillourokambos^3^. So there is reason to expect a bias towards hunted animals, with respect to fused calcanei, since managed suids are often culled prior to 24 months. Nonetheless, these results suggest that at least a small part of the suids exploited for their meat had been penned. Such practice is supported by the occurrence of curvilinear trenches—interpreted as fences^62^—discovered in the early phases of the PPNB occupation of Shillourokambos (10,500–10,200 cal. BP). These could have been used to limit the mobility of valuable livestock, including pigs, and protect cultivated plots from being ravaged. It is also supported by (1) a general decrease of most of the post-cranial classical osteometric measurements throughout the middle and late Cypro-PPNB of Shillourokambos, indicative of a domestication process similar to the one on the continent^10^; (2) the paleodemographic evidence of seasonal culling typical of husbandry practices^62^ and (3) an increasing abundance (25 bones in total), from the middle to the late phases of Shillourokambos, of an equal proportion of prenatal (90 gestation days) and neonatal (1–2 weeks after birth) bones, suggesting both the presence of pregnant sows and birthing in the village, and the emergence of typical early herding abortifacient pathologies and neonatal mortality^10^. Along with these observations, 3D morphometric evidence suggests that the human exploitation of *Sus scrofa*, at least towards the end of the PPNB in Cyprus, could have relied on the seasonal culling of suids living under anthropogenic control.

Finally, the morphological stasis of *Sus scrofa circeus* during twenty centuries, from the Late Cypro-PPNA up to the Late Cypro-PPNB suggest that animal management in the PPNB did not coincide with the introduction of domestic pigs from the mainland, otherwise the dental form in pigs would have diverged from that recorded in local Cypriot wild boar predating this cultural event. Other osteoarchaeological observations at Shillourokambos demonstrate that suid husbandry started as early as the Cypro-PPNB (c. 10,000 cal. BP) or even slightly earlier^3,10^. Our results indicate that it resulted from a local domestication of the Cypriot wild boar, introduced by Epipaleolithic people to the island 2500 years earlier, which were immediately released into the wild and hunted as the only large game for twenty-five centuries. Apart from its timing, this scenario is very similar to the one of goat domestication in Cyprus: early domestic goats were introduced to the island between 10,500 and 10,000 cal. BP, then immediately, or very soon thereafter, released into the wild. These feral goats were hunted for c. 500 years, before being finally locally domesticated c. 9,500 BP, at the turn of the Middle and Late Cypro-PPNB^3,4,7^.

The aforementioned scenarios cannot be generalized for all ungulates transferred to Cyprus. Domestic cattle and sheep, introduced broadly contemporaneously, were husbanded from their introduction to Cyprus onwards^3^, whereas Mesopotamian fallow deer were released into the wild immediately following their introduction and were never domesticated at all^8^. However, the wild boar and goat scenarios strengthen the idea that the PPN societies of Cyprus witnessed similar dynamics as their homologues on the nearby mainland, who first initiated the management and domestication of ungulates, thus giving birth to multiple domestic breeds found today across the globe. This confirms that the insular location of Cyprus, situated some 70–80 km offshore at that time, did not represent a strong cultural barrier^6^ and that Cyprus actively participated in the vast South-West Asian Neolithic core area, where the first societies of farmers emerged more than 10,000 years ago.
