# Author Correction: Bones geometric morphometrics illustrate 10th millennium cal. BP domestication of autochthonous Cypriot wild boar (*Sus scrofa circeus* nov. ssp)

**DOI:** 10.1038/s41598-021-96921-4

**Published:** 2021-09-22

**Authors:** Thomas Cucchi, Auriale Domont, Hugo Harbers, Allowen Evin, Roger Alcàntara Fors, Maria Saña, Charlotte Leduc, Aurélie Guidez, Anne Bridault, Hitomi Hongo, Max Price, Joris Peters, François Briois, Jean Guilaine, Jean‑Denis Vigne

**Affiliations:** 1 Archéozoologie, Archéobotanique: Sociétés, Pratiques et Environnements, AASPE UMR 7209, CNRS/Muséum National d’Histoire Naturelle, Paris, France; 2 ISEM, University of Montpellier, Montpellier, France; 3 Autonomous University of Barcelona, Barcelona, Spain; 4 Trajectoires, de la Sédentarisation à l’État, UMR 8215, Maison de l’Archéologie et de l’Ethnologie, 21 Allée de l’Université, 92000 Nanterre, France; 5 Archimède, Archéologie et Histoire Ancienne: Méditerranée Europe, UMR 7044, Université de Strasboug, Strasboug, France; 6 ArScAn, Equipe Archéologies Environnementales, UMR 7041, CNRS, Maison de l’Archéologie et de l’Ethnologie, 21 Allée de l’Université, 92000 Nanterre, France; 7 Department of Evolutionary Studies of Biosystems, School of Advanced Sciences, Graduate University for Advanced Studies, Shonan Village, Hayama, Kanagawa 240‑0193 Japan; 8 Department of Materials Science and Engineering, Massachusetts Institute of Technology, 77 Massachusetts Avenue, Cambridge, MA 02143 USA; 9 ArchaeoBioCenter and Institute of Palaeoanatomy, Domestication Research and the History of Veterinary Medicine, LMU Munich, 80539 Munich, Germany; 10 SNSB, State Collection of Anthropology and Palaeoanatomy, 80333 Munich, Germany; 11 EHESS, UMR 5608, Travaux et Recherches Archéologiques sur les Cultures, les Espaces et les Sociétés (TRACES), Université Jean Jaurès, Toulouse, France; 12 Collège de France, 11, Place Marcelin‑Berthelot, 75005 Paris, France

Correction to: *Scientific Reports* 10.1038/s41598-021-90933-w, published online 01 June 2021

Allowen Evin, Roger Alcàntara Fors and Maria Saña were omitted from the author list in the original version of this Article.

The Author Contributions section now reads:

“T.C. designed the research and wrote the paper in collaboration with J.D.V., J.P. and M.P. A.D. and H.Ha. collected and analysed respectively the dental and calcaneus morphometric dataset. M.P., A.E., R.A.F., and M.S., provided access to image collections of molars from modern and archaeological samples. A.G., A.B., C.L., H.Ho., J.P. and M.P. provided contextualised archaelogical samples. J.D.V., J.B. and J.G. excavated Klimonas and Shillourokombos. All authors reviewed the manuscript.”

Furthermore, the Acknowledgements section in the original version of this Article was incomplete. It now reads:

“This research has been funded by the ANR, through the Domexp project (ANR-13-JSH3-0003-01); LabEx ANR-10-LABX-0003-BCDiv, in the ‘Investissements d’avenir’ programme ANR-11-IDEX-0004-02 and project Emergence SU-19-3-EMRG-02. This research has also benefited from financial supports of the Muséum national d’Histoire naturelle (Paris) and the CNRS INEE (Institut écologie et environnement; SEEG “Limassol”). It also received support of the French School at Athens, the French Ministère des affaires étrangères and the Department of Antiquities of the Republic of Cyprus. DFG-funding to J. Peters (PE 424/10, 1-4) to study PPN archaeofaunas in Southeast Anatolia is gratefully acknowledged. Roger Alcàntara Fors and Maria Saña have been supported by the grants HAR2014-60081-R (PI Maria Saña), HAR2016-78416-P (PI Miquel Molist), and HAR2017-88304-P (PI Maria Saña). We are most grateful to Greger Larson and Keith Dobney for the financial support of the NERC project “Pigs, People and the Néolithisation of Europe” which allowed the trips for image acquisition by Allowen Evin, Joe Owen and Linus Girdland Flink in the Natural History Museum of Berlin and in the Department of Archaeology of Istanbul. We are most grateful to Frieder Mayer for giving Allowen Evin access to the wild boar collections of the Natural History Museum of Berlin. We would like to thank Marie Balasse and Delphine Frémondeau for collecting skulls of Corsican wild boars in collaboration with the INRAE of Corte. We are most thankful to Stéphanie Bréhard and Rose-Marie Arbogast for providing access and contextual information respectively on the Gazel and Arconciel Mesolithic samples.”

In addition, the original version of this Article contained errors in the Reference List, where references [43] ‘Groves, C. P. *et al.* Current views on taxonomy and zoogeography of the genus *Sus. Pigs Hum.* **10**, 15–29 (2007).’ and [52] ‘Jean-Denis, V. The large «true» Mediterranean Island as a model for the Holocene human impact on the European vertebrate fauna? Recent data and new reflections. *Holocene Hist. Eur. Vertebr. Fauna Mod. Asp. Res. Berl. Atelier* 295–322 (1999).’ were added erroneously. These references have been removed, and the Reference List has been renumbered.

The original Article has been corrected.

